# Cerebral infarction presenting with bilateral sudden deafness as the primary symptom: a case report

**DOI:** 10.1177/03000605241305483

**Published:** 2024-12-23

**Authors:** Jie Zhang, Lin Zhou, Tao Liu, Yuhan Cheng, Yanling Dou

**Affiliations:** 1 Department of Otorhinolaryngology Head and Neck Surgery, Western Theater Air Force Hospital of PLA, Chengdu, China; 2 Department of Neurology, Meishan Pengshan District Traditional Chinese Medicine Hospital, Meishan, China

**Keywords:** Sudden deafness, cerebral infarction, early diagnosis, primary symptom, case report, basilar artery occlusion, stenosis

## Abstract

The current case report presents a rare occurrence of cerebral infarction with bilateral sudden deafness as the primary symptom. The patient was a 59-year-old man with hypertension who tested positive for new coronary antibodies and had a long history of smoking and alcohol consumption. Despite receiving treatment for sudden deafness, the patient’s condition rapidly deteriorated and he was diagnosed with basilar artery occlusion and stenosis. The patient died 5 days after mechanical recanalization of the artery. Sudden binaural deafness is a rare clinical condition that may be a prodromal symptom of brainstem infarction. Early diagnosis and prompt treatment are essential for reducing mortality and disability.

## Introduction

Sudden deafness is a condition characterized by a sudden loss of hearing of unknown origin that occurs within 72 hours, with a hearing loss ≥20 dB hearing level in at least two adjacent frequencies.^1,2^ The etiology and pathogenesis of sudden deafness remain unclear. Sudden deafness may be related to local or systemic factors such as vascular lesions, viral infections, autoimmune diseases, infectious diseases, and tumors. Moreover, the possible pathogenesis of sudden deafness may include vasoconstriction or inner ear dysfunction, vascular embolism or thrombosis, accumulation of membrane vagus fluid, and hair cell injury.^3^ Although cerebral infarction with unilateral hearing loss as the primary manifestation is relatively common, cerebral infarction causing bilateral sudden deafness is rare.^4,5^ If bilateral profound deafness occurs, it often indicates a poor prognosis.^6^ In the current report, we present the case of a patient with bilateral basilar artery occlusion and stenosis who had bilateral sudden deafness as the first manifestation, and analyze the clinical course.

## Case presentation

**Figure 1.**
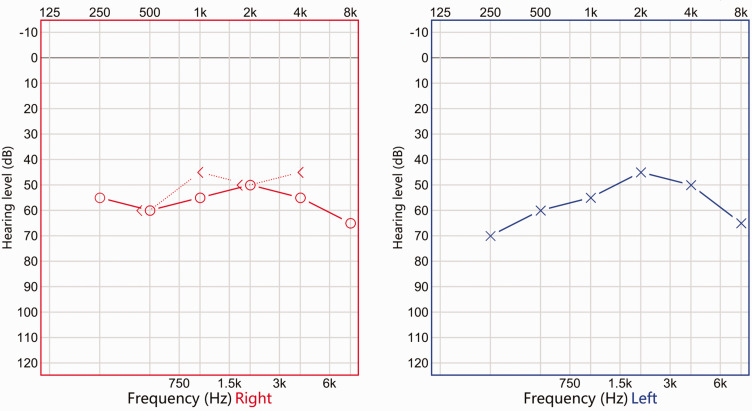
Pure tone audiometry at the time of the patient's admission to hospital.

A 59-year-old man was admitted to the hospital with sudden deafness in both ears and tinnitus for 1 day, which was accompanied by dizziness without nausea or vomiting. An audiological examination revealed moderate sensorineural hearing loss in both ears (Figure 1). Two months earlier, hypertension had been detected during a physical examination but was left untreated. The patient’s blood pressure at the time of admission was 160/102 mmHg. One week earlier, the patient had tested positive for new coronary antibodies. He had a long history of smoking and alcohol consumption, smoking more than 10 cigarettes a day and drinking approximately 100 mL of alcohol each time (converted values: approximately 0.12 g nicotine per day and 31.56 g pure alcohol per drinking session). He had been drunk the day before the disease onset.

The patient's condition rapidly deteriorated, and he received methylprednisolone (40 mg) injections and *Ginkgo biloba* extract (87.5 mg) by intravenous drip, following guidelines for the treatment of sudden deafness.^2,7^ His blood pressure was also controlled with olmesartan (20 mg per day). A head computed tomography (CT) scan revealed no abnormalities. However, on day 3 of admission (35 hours after admission), the patient suddenly became unconscious after toileting, with obvious sputum sounds, and his oxygen saturation dropped below 70%. The patient was immediately intubated with balloon-assisted breathing and his oxygen saturation rose to approximately 90%. However, the patient remained unconscious and had unequal pupils (6 mm on the left and 2 mm on the right); he was therefore transferred to the intensive care unit.

**Figure 2.**
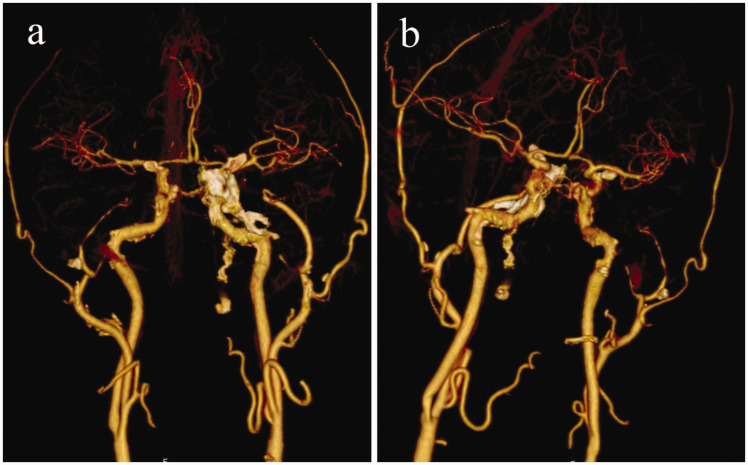
Computed tomography angiography images. (a, b) Images showing mixed plaque formation in the V4 segment of the right vertebral artery, combined with mild stenosis of the vessel lumen; soft plaque formation in the V2–V4 segments of the left vertebral artery, combined with occlusion of the vessel lumen; soft plaque formation in the middle and distal segments of the basilar artery, combined with occlusion of the vessel lumen; and mixed plaque formation in the P1 segment of the left posterior and right superior cerebellar arteries, with severe stenosis of the vessel lumen.

**Figure 3.**
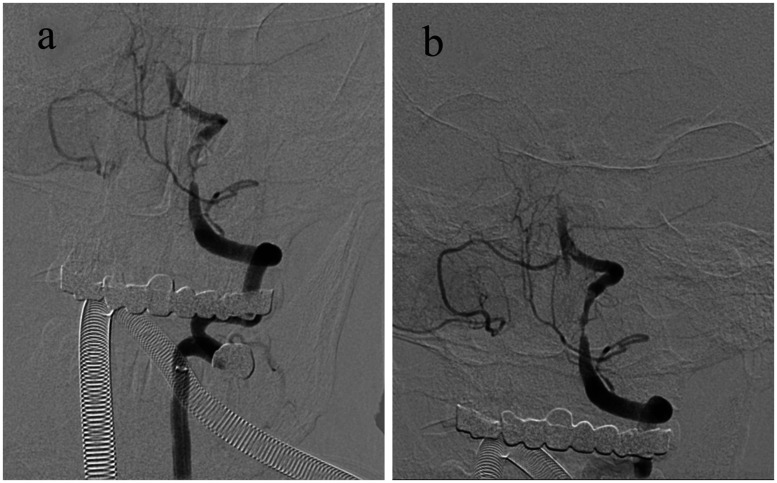
Cerebral angiography images. (a) Before surgery, there was a stenosis in the left vertebral artery and (b) during the operation, a partial thrombus was removed from the left vertebral artery. However, imaging of the left vertebral artery showed poor results, and the distal end of the basilar artery was unable to be observed.

The results of CT angiography (CTA) demonstrated mixed plaque formation in the V4 segment of the right vertebral artery, combined with mild luminal stenosis; soft plaque formation in the V2–V4 segment of the left vertebral artery, combined with luminal occlusion; soft plaque formation in the middle and distal segments of the basilar artery, combined with luminal occlusion; and mixed plaque formation in the P1 segment of the right superior and left posterior cerebral arteries, with severe luminal stenosis (Figure 2(a), (b)). The patient's family did not agree to thrombolysis, and mechanical recanalization with cerebral angiography of the basilar artery was therefore performed. Although some of the thrombus was removed from the left vertebral artery, the distal part of the basilar artery was not apparent (Figure 3(a), (b)). Intraoperatively, a small amount of extravasated contrast was observed in the blood supply area of the basilar artery, and the procedure was stopped because of the risk of bleeding.

**Figure 4.**
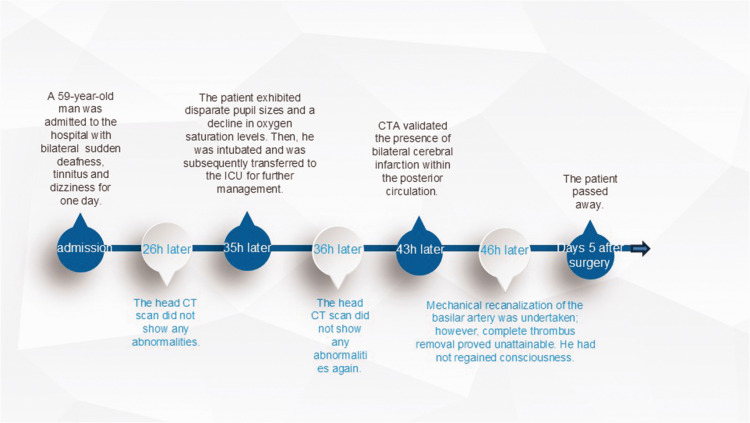
Disease progression timeline. CT, computed tomography; CTA, computed tomography angiography; h, hours; ICU, intensive care unit.

The patient did not regain consciousness after the operation, and he required ventilator support and high doses of vasoactive drugs to maintain his blood pressure. On day 5 after surgery, the patient died. The disease progression timeline is shown in Figure 4.

All of the patient’s specific information has been de-identified. The patient’s family signed an informed consent form in which they agreed to the publication of the case and associated images. The case report was approved by the Ethics Committee of Western Theater Air Force Hospital of PLA (approval number: 2024018). The reporting of this study conforms to CARE (CAse REports) guidelines.^8^

## Discussion

Bilateral sudden deafness is a rare clinical condition characterized by an acute loss of hearing in both ears that occurs simultaneously or sequentially in a short period of time. It accounts for only 1.7% to 8.6% of cases of sudden deafness and can be caused by various factors including vascular disease, poisoning, infection, and genetic factors.^9–12^ Bilateral sudden deafness is associated with more severe hearing loss and poorer healing than unilateral sudden deafness.^13^

When bilateral sudden sensorineural hearing loss is present, it is necessary to investigate the presence of underlying systemic disease. Lee et al.^14^ reported that sudden bilateral deafness may be a prodromal symptom of brainstem infarction, and that vertebrobasilar artery occlusive disease should be considered in the differential diagnosis of this condition. However, stroke and transient ischemic attack rarely present as isolated sudden deafness, and the presence of new focal neurological symptoms or signs in patients with sudden deafness indicates central nervous system involvement.^1,15^

The inner ear is particularly vulnerable to ischemia because of its small size and the tortuous nature of the arteries that supply it, which have slow blood flow and few collateral branches. Ischemic infarction of the anterior inferior cerebellar artery is generally associated with vertigo, hearing loss, facial palsy, nystagmus, and ataxia, and is often accompanied by other brainstem or cerebellar symptoms.^16,17^ The labyrinthine artery is not substantially supplied by collateral circulation. Unlike many other vessels, it relies primarily on its direct origin from the anterior inferior cerebellar artery or basilar artery.^18^ It therefore lacks robust alternative pathways to compensate for any occlusion or compromise. The corresponding onset of deafness is relatively isolated and severe, and persistent hearing loss may be caused by ischemia to the inner ear.^12^

The rapid progression of our patient’s disease was closely linked to his vertebrobasilar artery malformation. As revealed by CTA, there was bilateral vertebral basilar artery plaque occlusion and luminal narrowing. However, it is essential to mention vertebrobasilar dolichoectasia (VBD), which can also cause audio-vestibular manifestations.^19^ Independent risk factors for VBD include male sex, old age, hypertension, smoking, and a history of myocardial infarction. VBD, in turn, serves as an additional independent risk factor for stroke alongside old age, hypertension, and diabetes. However, VBD is a rare condition, and its symptoms primarily result from cerebral nerve compression as a result of blood vessel alterations.

In recent years, cortical deafness—a type of bilateral central hearing loss—has often been mentioned. Patients with cortical deafness retain brainstem auditory-evoked potentials, lose the perception of non-verbal and verbal sounds, show no response to all auditory stimuli, and have abnormal pure tone hearing test results; cortical deafness is thus considered the most serious kind of cortical hearing impairment.^20^ Early diagnosis and prompt treatment are essential for reducing the mortality and disability caused by cerebral infarction with sudden deafness as the first manifestation.^21^

A neurological examination of our patient did not reveal any definitive abnormalities at the time of admission. However, the patient had experienced hearing loss in both ears with tinnitus and dizziness for 1 day and was within 1 week of the appearance of sudden deafness. According to the *Guideline of Diagnosis and Treatment of Sudden Deafness (2015)*,^2^ a magnetic resonance imaging (MRI) examination is not recommended within 1 week of onset, except when there is high suspicion of stroke. The *Chinese Guidelines for Diagnosis and Treatment of Acute Ischemic Stroke 2018*^22^ recommend plain cranial CT as the preferred test for stroke diagnosis. Our patient therefore underwent a cranial CT scan at the time of admission and once again after the onset of severe cerebral infarction; however, no signs of cerebral infarction were detected. A diagnosis of cerebral infarction was eventually confirmed by CTA, which took some time to complete.

Current guidelines for sudden deafness or stroke recommend imaging techniques such as CT or MRI to diagnose a cerebral infarction as soon as possible after the onset of symptoms. However, these imaging methods may not always be adequate for the early identification of cerebral infarction. CTA and magnetic resonance angiography (MRA) are two imaging methods that offer relatively good visualization of the responsible vessel and can detect lesions such as vessel blockage or stenosis. These methods are useful for the early identification of the vessel responsible for cerebral infarction, and for further revascularization treatment. However, the question of when to intervene with CTA and MRA requires further discussion.

Generally, CTA or MRA can be considered when a patient has conflicting clinical symptoms and imaging manifestations, or when the diagnosis is uncertain. Furthermore, CTA or MRA screening may be useful for the early detection of vascular lesions in high-risk groups (e.g., patients with hypertension, diabetes, and/or hyperlipidemia) or in patients who have already had a stroke. However, it is important to note that CTA carries some risk from radiation and contrast agents, and MRA has safety implications for patients with foreign ferromagnetic bodies. The use of these techniques should therefore involve the careful consideration of their benefits and risks. Furthermore, in most tertiary hospitals in China, CTA and MRA are not emergency examinations and require an appointment. The development of a perfect medical emergency system is therefore required because, for patients with acute cerebral infarction, “time is brain.” Effectively shortening the time from onset to treatment and from onset to examination is a problem that urgently needs solving.

The etiology of acute cerebral infarction includes large artery atherosclerosis, cardiogenic embolism, small artery occlusion, other causes, and unknown causes. Banerjee et al.^23^ reported that the atherosclerotic type of large arteries accounts for the highest proportion of deaths in acute cerebral infarction, and this number is increasing over time. The number of patients with acute cerebral infarction is also increasing over time. Risk factors such as advanced age, hypertension, hyperlipidemia, diabetes mellitus, smoking, and alcohol consumption may promote the appearance of large-artery atherosclerotic cerebral infarction.

In our patient, cranial CTA and digital subtraction angiography indicated multiple plaques, stenosis, and occlusion of the vertebrobasilar artery. The patient also had previously untreated hypertension and a history of long-term smoking and alcohol consumption, and had been drunk 1 day before admission. In such a case, other cerebral infarcts, such as cardiogenic infarcts, are therefore less likely, whereas large atherosclerotic cerebral infarcts are more likely.

The basilar artery is the main source of blood supply to the brainstem, which is a respiratory and circulatory center. Brainstem infarction often has acute onset, a critical condition, poor recovery of clinical symptoms, and a high mortality rate. Our patient had multiple plaques, stenosis, and occlusion of the vertebrobasilar artery, with poor collateral circulation in vessels. Considering the clinical symptoms and risk factors of the patient, the possibility of a brainstem infarction was high. In the treatment of acute cerebral infarction, timely and effective revascularization and reperfusion are crucial.^24,25^

Intravenous thrombolysis has the advantages of low invasiveness, a low price, easy access, and an easy operation; however, it often fails to achieve the desired therapeutic effect because of time limitations, a low revascularization rate, and the relative ease of recurrent vascular occlusion. As a newer method of vascular intervention, arterial thrombectomy allows surgeons to effectively observe the revascularization state. It can also provide opportunities for revascularization in patients who miss the intravenous thrombolysis time window or have a poor outcome with intravenous thrombolysis. However, it also has limitations, such as being a relatively expensive and complex operation. Individual treatment modes should therefore be selected by assessing the situation of each patient.

Revascularization modalities include mechanical retrieval, direct aspiration techniques, and angioplasty (balloon dilation and/or stenting). A study by Huo et al.^26^ revealed that aspiration is recommended for patients with acute basilar artery tip occlusion. The presence of risk factors such as bleeding, ischemia–reperfusion injury, plaque dislodgement, vasospasm, and vasoneurological reaction should be taken into account when considering the effective opening of the vessel and salvage of the ischemic infarcted area. Treatments related to complications in patients with cerebral infarction are also very important for patient prognosis. Patients with brainstem infarction often have complications such as pulmonary infection, electrolyte disorders, and hypoproteinemia because of a poor state of consciousness, weak swallowing function, prolonged bed rest, and difficulty in sputum evacuation. These complications can worsen the prognostic outcome of patients. Moreover, patients with an insidious onset, poor vascular base, and infarct site in a core region are relatively likely to have rapid progression, critical condition, and eventual death.

In conclusion, sudden deafness guidelines may not be sufficient for the early identification of cerebral infarction with sudden deafness as the first manifestation. The timing of CTA, MRI, and digital subtraction angiography-related examinations depends on each patient’s clinical presentation and medical history. Patients with symptoms of cerebral infarction or chronic diseases such as hypertension, diabetes, or hyperlipidemia should undergo CTA, CT perfusion, MRA, and digital subtraction angiography as soon as possible if CT and MRI alone cannot confirm a diagnosis. This may help to determine the presence and location of cerebral infarction and allow for early therapeutic measures.
